# Post-Operative Bleeding Complications in a Periodontitis Patient Testing Positive for COVID-19

**DOI:** 10.3390/dj10060110

**Published:** 2022-06-14

**Authors:** Georgios Loukas, Madeline X. F. Kosho, Spiros Paraskevas, Bruno G. Loos

**Affiliations:** Department of Periodontology, Academic Centre for Dentistry, Amsterdam (ACTA), University of Amsterdam and Vrije Universiteit Amsterdam, 1081 LA Amsterdam, The Netherlands; m.kosho@acta.nl (M.X.F.K.); s.paraskevas@acta.nl (S.P.); b.loos@acta.nl (B.G.L.)

**Keywords:** COVID-19, periodontal surgery, bleeding complications

## Abstract

Recent scientific evidence states that a subset of COVID-19 patients may have a risk of increased bleeding tendency. This case report presents a healthy 38-year-old woman with generalized stage III, grade C periodontitis with an abnormal post-operative blood clot formation who tested positive for COVID-19 after a standard periodontal surgery. Previously, two periodontal surgeries proceeded without any complications and were considered standard. On day one after the third periodontal surgery the patient had no complaints. On day two the patient reported excess bleeding in the oral cavity from the operated area simultaneously with fever and loss of taste. On day three the patient was seen in our clinic; general malaise symptoms and bleeding tendency had started to decline and the patient received a COVID-19 PCR test. At day four the test resulted positive, and she reported no further complaints of intraoral bleeding. Six months later the taste of the patient was still distorted. For this patient with free medical anamnesis, we suggest that the patient had increased plasma levels of tissue plasminogen activator during the crucial postoperative period due to an acute COVID-19 infection. This led to increased plasmin levels with a hyper-fibrinolytic state as a consequence.

## 1. Introduction

In December 2019, an outbreak of pneumonia of unknown etiology occurred in Wuhan, China, which resulted in the isolation of a new coronavirus strain. Coronavirus disease 2019 (COVID-19) is, according to the World Health Organization, an infectious disease caused by the SARS-CoV-2 virus. Since its emergence, aside from the death toll, COVID-19 has also had a remarkable disruptive impact on the health care system globally, with many consequences also in the field of dentistry [1]. Since the behavior of the virus during the so-called “first wave” was not elucidated, various necessary interventions like strict quarantine kept individuals away from dental care [2].

With the world witnessing the fast development of vaccines and their provided immunogenicity, the scientific community within dentistry turned its scope towards the oral manifestations of COVID-19 [3]. Since it was demonstrated that SARS-CoV-2 can be detected in saliva [4] and calculus [5] and with dysgeusia [6] being one of the first recognized oral symptoms of COVID-19, several ramifications of the virus in the oral cavity were investigated [7,8]. One of these ramifications is the association with COVID-19 and periodontitis [9].

Periodontitis is a chronic inflammatory disease of the supporting tissues of the teeth [10]. In this disease, inflammatory reactions cause the destruction of connective tissue, periodontal ligament, and alveolar bone. As a result, deep periodontal pockets appear, containing a dysbiotic microbiome and dental calculus. It is well known that patients with untreated severe periodontitis present with an inflamed surface area of 5–20 cm^2^ [11,12]. Interestingly, recently it was found that periodontitis is associated with severe COVID-19; patients with severe periodontitis have higher risk of COVID-19 complications like ICU admission (OR = 3.54), the need for assisted ventilation (OR = 4.57), or death (OR = 8.81) [9]. Somewhat comparable results were found recently in a pilot study [13]. However, in the latter paper the association of periodontal breakdown with COVID-19 severity lost significance when corrected for age, sex, and BMI. In a post-mortem study, genetic material from SARS-CoV-2 was found present in the periodontium [14], and another pilot study presented a positive association of deleterious oral health-related conditions, especially periodontitis, and severe COVID-19 outcomes in hospitalized COVID-19 patients [15].

In general, periodontitis can be successfully controlled, and teeth can be retained for life [16]. In order to reach this level of periodontal homeostasis, patients undergo an initial, non-surgical treatment phase. If the periodontal response to this phase is good but residual inflammation and residual deepened periodontal lesions (≥5 mm) are still present, the patient can enter an adjunctive surgical phase. Nowadays, the clinician performing the periodontal surgeries has an abundance of arrows in his quiver to tackle various problems and to approach a spectrum of desirable results [17]. However, regardless of the type of periodontal surgical intervention, a stable blood clot and consequently optimal post-operative healing in the first days is obviously always necessary [18,19,20]. Patients with no genetic, autoimmune, or acquired bleeding susceptibility may present with bleeding complications after a periodontal surgery if they fail to follow the clinician’s postoperative instructions. Additionally, a clinician’s negligence to thoroughly investigate a patient’s medical anamnesis, mishandling of tissues, engaging with major vessels, and poor primary closure of the wound could lead to post-operative hemorrhage [21].

Acutely ill patients who experience SARS-CoV-2 infection have increased risk of arterial and venous thrombosis [22]. However, recent findings indicate that aside from this heightened clotting risk, some COVID-19 patients have an unbalanced fibrinolytic homeostasis [23,24]. This subset of COVID-19 patients with a hyper fibrinolytic state could be of high importance in the field of periodontology and oral surgery, especially in the early post-operative days after various oral surgical procedures.

The aim of this case report is to present a 38-year-old woman with generalized stage III, grade C periodontitis [25] with a distorted post-operative blood clot formation who tested positive for COVID-19 after a periodontal surgery.

## 2. Case Report

### 2.1. Intake

A 38-year-old woman was referred from a private dental practice in Amsterdam to the Department of Periodontology, Academic Centre for Dentistry Amsterdam (ACTA), University of Amsterdam and Vrije Universiteit Amsterdam, Amsterdam, the Netherlands. The reason for the referral was “treatment of periodontitis.” At the intake, the patient’s complaints were: “I have problems with my gums” and her expectations were: “I want a healthy mouth and an orthodontic treatment.” A medical anamnesis questionnaire was sent to the patient to be filled in prior to the appointment, and in combination with an interview for the patient’s health at the intake, the self-reported medical record was verified. Furthermore, the patient was not receiving any medication, had never smoked (0 packs/year), and her body mass index (BMI) was 26.6 kg/m^2^.

**Figure 1:**
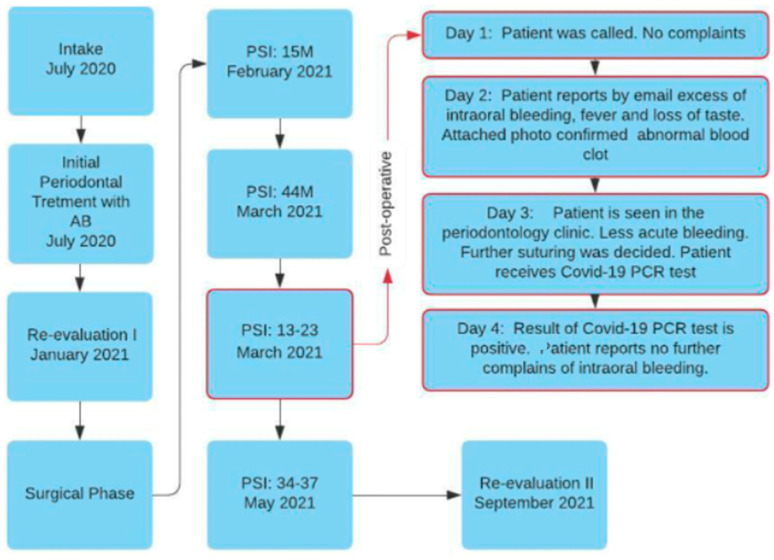
Treatment modalities from intake to the end of active periodontal treatment at 6-month post-operative. Abbreviations: AB = adjunctive use of antibiotics (amoxicillin, 375 mg, and metronidazole, 500 mg) three times a day for 7 days starting 1 day prior to the intervention, PSI = periodontal surgical intervention with involved tooth number(s), M = mesial.

**Figure 2:**
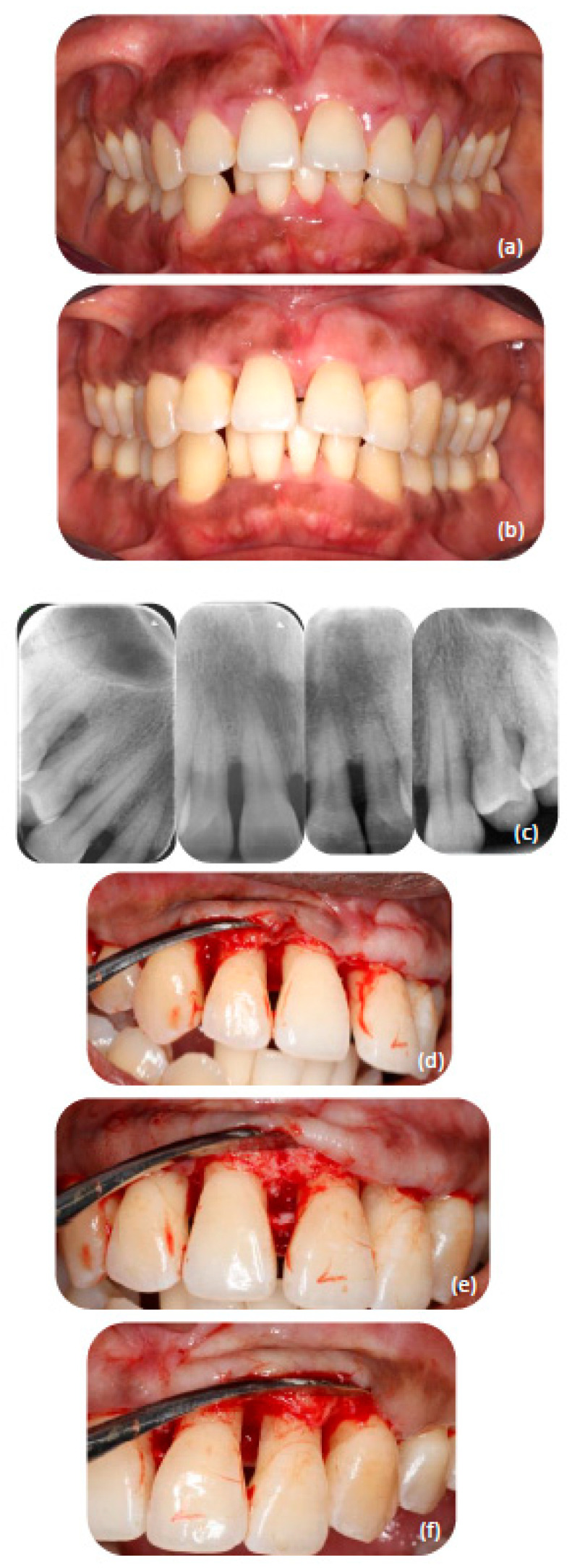
(**a**) Intraoral view at intake. (**b**) Intraoral view at 6 months after initial non-surgical treatment. (**c**) Radiographic overview of the maxillary anterior area. (**d**–**f**) Intraoral view during surgery.

A complete overview of all periodontal treatment for this patient can be seen in the flowchart (Figure 1). At the intake, an intraoral examination revealed no peculiar abnormalities. Periodontal intraoral examination revealed natural pigmentation, generalized swelling with redness at the free gingiva, and lack of pointy architecture at the interdental papillae, especially at the interdental diastemas at the anterior areas (Figure 2a). Apart from third molars, a complete natural dentition was present. 

### 2.2. Initial Non-Surgical Periodontal Treatment

Charting of the periodontal parameters revealed a full-mouth bleeding score (FMBS) on probing of 96%; a full-mouth plaque score (FMPS) of 62%; generalized negative recessions and deepened pockets ≥6 mm; localized suppuration at 13, 23, and 31; furcation involvement [26] at 17, 16, 26, 27, 37, and 47; and mobility I [27] for 12, 22, 32, 31, and 42. Furthermore, localized supragingival calculus was found in the lower front lingual, but no subgingival calculus was felt during probing.

Radiographic evaluation revealed a non-horizontal alveolar bone loss pattern with angular defects and with 22 teeth showing alveolar bone loss extending to the mid-third of the root or beyond. Radiographic data from 2014 and 2015 revealed the progression of alveolar bone loss, especially at the sites with the angular bony defects.

The abovementioned findings led us to a diagnosis of a 38-year-old woman with generalized stage III, grade C periodontitis [25]. In the initial non-surgical periodontal treatment phase, a full mouth disinfection protocol was performed within 48 h [28,29], with adjunctive simultaneous use of two antibiotics (amoxicillin, 375 mg, and metronidazole, 500 mg). Additionally, the patient was instructed to rinse with chlorhexidine 0.12% and peroxide 3% for 14 days starting the day of the intervention. The patient was recalled until re-evaluation of the oral hygiene was performed, which was reinforced when needed.

At 6 months post-non-surgical periodontal treatment, an evaluation of the periodontal parameters was performed. The periodontal tissues had responded favorably (Figure 2b) with generalized reduction in pocket depths and FMBS and FMPS reduced to 19% and 8%, respectively. Generalized recessions were measured and the results indicated clinical attachment gain in numerous places. Moreover, new dental radiographs showed areas with alveolar bone filling in angular bony defects.

### 2.3. Surgical Phase

Following the results of the initial periodontal treatment, four periodontal surgical interventions (PSI) were planned with the patient’s consent:

(I) PSI: Modified papilla preservation, including an attempt to regenerate with amelogenin (Enamel Matrix Derivative) at the residual deep intrabony defect (≥3 mm), mesial to 15; procedure according to Cortellini and Tonetti [30].
(II) PSI: Modified papilla preservation, including an attempt to regenerate with amelogenin (Enamel Matrix Derivative) at the residual deep intrabony defect (≥3 mm), mesial to 44; procedure according to Cortellini and Tonetti [30].
(III) PSI: Accessing flap 13–23 with a combination of buccal papilla preservation at 11–21 and osteoplasty where needed (Figure 2d–f); procedure is a combination of the modified papilla preservation technique as described by Cortellini and Tonetti [30] and open flap debridement as described by Carranza and Takei [31].
(IV) PSI: Shortening flap 34–37; procedure according to Carranza and Takei [31].

All four of the surgical procedures were performed by a postgraduate student (GL) specializing in periodontics and implant dentistry in the Department of Periodontology, ACTA. The postgraduate student was seconded with active chair-side supervision from a very experienced periodontist (SP), a staff member, and a clinical instructor of the periodontology specialty program in ACTA.

### 2.4. Post-Operative Bleeding Complications

Periodontal surgical procedures (I) and (II) proceeded without any complications and were considered standard.

The surgical intervention (III) had a twofold purpose: firstly, to eliminate residual pockets, and secondly, to inspect the crestal bone architecture. Osteoplasty was performed in discrepancies (Figure 2b–d). Since the patient expressed her interest in improving her smile with an orthodontic treatment after the end of the active periodontal phase (4 mm overjet of the upper incisors and interdental diastemas), an optimization of the upper anterior area from a periodontal perspective was necessary.

**Figure 3:**
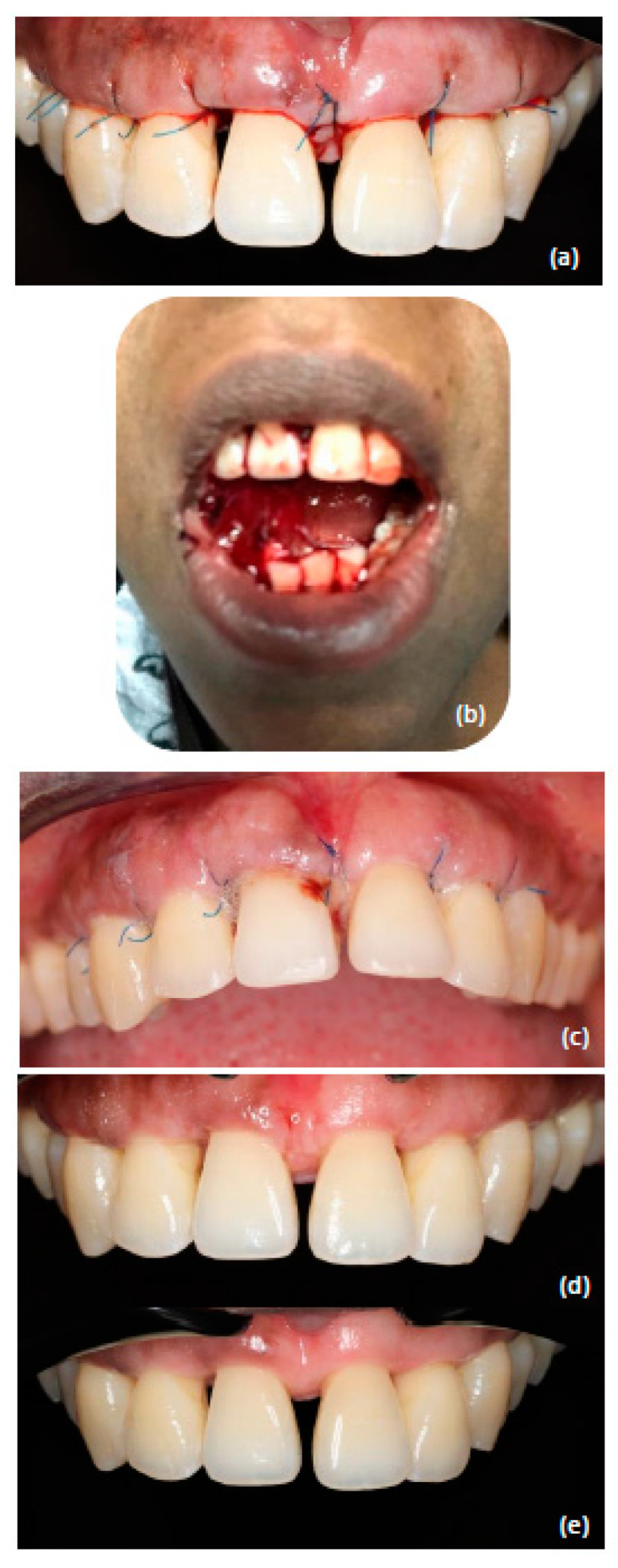
(**a**) Direct post-operative intraoral view. (**b**) Intraoral view as sent by the patient on day 2. (**c**) Intraoral view on day 3. (**d**) Intraoral view 3 weeks post-operative immediately after suture removal. (**e**) Intraoral view 6 months post-operative at the re-evaluation of the surgical phase.

During the intervention nothing out of the norm was noticed. Although during this particular procedure we performed some routine osseoplasty in the interdental areas, no intrabony vessels were severed and no excessive bleeding was noticed. Furthermore, the soft and hard tissues were respected, and we gave special attention during palatal elevation to not severe the tissues of the papilla incisiva. Degranulation of the interdental areas was performed in combination with bone corrections by osteoplasty and thinning of the tissues. The tissues were rinsed thoroughly with sterile 0.9% sodium chloride and eventually sutured. Clinical pictures were taken during the procedure and after suturing (Figure 3a). Pressure was applied to the operated tissues with gauze dipped in sodium chloride solution. The patient was given oral and written post-operative instructions. After 10 min the wound was inspected again and showed no signs of bleeding.

On day one after this periodontal surgery (III) following our clinic’s protocol, the patient was called for a post-operative telephonic control. The patient had no complaints.

On day two post-operative, early in the morning, the patient reported by email excessive bleeding in the oral cavity from the operated area, simultaneously with fever and loss of taste. A photo taken by the patient confirmed the abnormal blood clot (Figure 3b). On day three, the patient was seen urgently in our clinic, yet her fever and general malaise had started to decline. During the appointment, the bleeding tendency was less acute than the day before (Figure 3c). Further suturing was decided on for prevention of possible additional bleeding. On the same day the patient received a COVID-19 PCR test and 24 h later, on day four, the report of the PCR was positive. At day four post-operative, the patient reported no further complaints of intraoral bleeding. In order to minimize the chances of possible spread of the virus, the patient was seen again after two weeks (three weeks post-operative) for suture removal (Figure 3d). The periodontal tissues exhibited a good healing pattern, as we had seen at the two previous surgeries. One month later, the last surgical intervention (IV) was performed, which was uncomplicated, and the early healing showed good epithelization in the interdental areas after 7 days.

**Figure 4:**
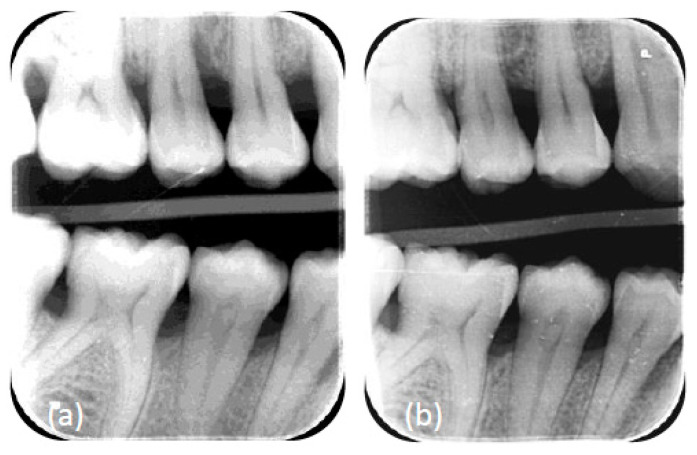
(**a**) Radiographic view of the angular defect 15 M before regenerative intervention. (**b**) Radiographic view of the angular defect mesial to 15 6 months after the intervention, attesting to a good healing capacity after periodontal surgery.

**Figure 5:**
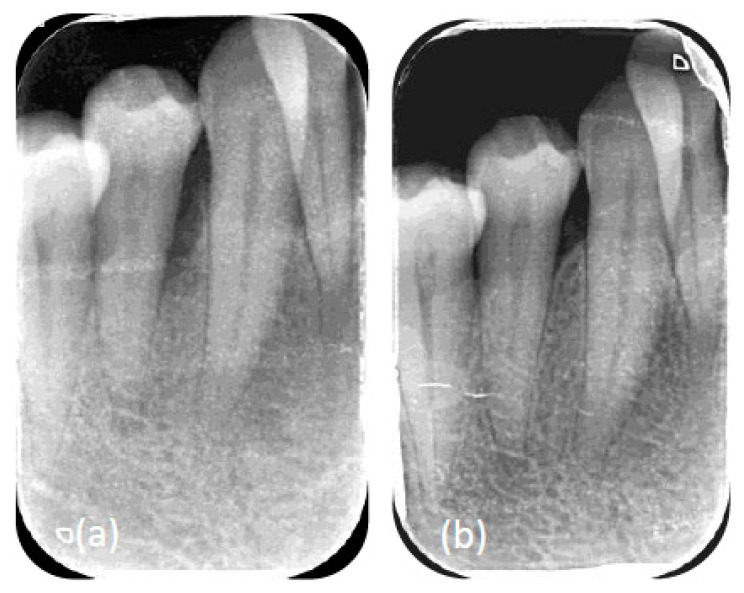
(**a**) Radiographic view of the angular defect 44 M before regenerative intervention. (**b**) Radiographic view of the angular defect mesial of the 44 6 months after the intervention, attesting to a good healing capacity after periodontal surgery.

At six months post-operative (Figure 3e), during the re-evaluation of the surgical phase, regarding COVID-19 complications, the patient reported that her taste was still distorted. From a periodontal standpoint, we had achieved the purposes of the periodontal treatment, having no residual bleeding pockets of ≥4 mm, an FMBS of 7%, and an FMPS of 3%. The surgeries with regenerative materials yielded remarkable bone fill in the intrabony defects (Figure 4 and Figure 5)

## 3. Discussion

In this report we present a case that showed post-operative bleeding after a periodontal surgical intervention. Since the patient presented without any specifics in her medical anamnesis and she showed uneventful healing, based on the literature [17], after two previous periodontal surgical interventions, how to explain this event is intriguing. In the case report we suggested that her simultaneous COVID-19 infection was the basis for this rare occurrence. We excluded the postoperative usage of aspirin, NSAID, or any other medication other than a limited usage of paracetamol (acetaminophen).

In an attempt to explain the current post-operative bleeding complication during an acute COVID-19 infection after the periodontal surgical procedure, we first should describe in brief normal wound healing as part of the healing process. After a surgical intervention, hemostasis takes place in the first 10–15 min [32]. Inflammation will be present for up to 72 h. The gradual proliferation of the gingival epithelium for complete wound coverage will last up to 21 days. The initial blood clot stimulates the growth of the epithelium with a pace of 0.5 mm per day [33], and for uncovered alveolar bone the surface epithelialization is complete after 7–14 days. During this process, the blot clot is gradually degraded through the process of fibrinolysis, and for sufficient wound healing in the first days, the fine-tuning between fibrinolysis and the pace of epithelization of the wound is of high importance. In general, this process is tightly controlled by the fibrin-rich thrombus itself, which becomes degraded by the protease plasmin. Plasmin is generated by the cleavage of the precursor plasminogen by plasminogen activators (PA) [34], both tissue-type (tPA) and urokinase-type (uPA). The principal inhibitor of the plasmin generation (i.e., inhibition of fibrinolysis) is plasminogen activator inhibitor-1 (PAI-1). As such, plasmin and PAI-1 play a key role in regulating fibrinolytic activity.

Regarding the cause of the observed post-operative bleeding in the current case during her COVID-19 infection, we can only speculate. A COVID-19-associated prothrombotic risk is known [35]; however, recent published data [24] present an enhanced bleeding risk complication in a subset of patients with active COVID-19. A potential mechanism that has been suggested involves increased levels of tPA (tissue-type plasminogen) during the acute phase of the COVID-19 infection, which generate excess levels of plasmin and increased fibrinolysis [23]. For their research [23], Zuo et al. collected plasma from hospitalized patients with COVID-19 and from healthy controls. Significantly higher levels of tPA and PAI-1 among patients with COVID-19 compared to the healthy controls were found, and more specifically for high levels of tPA, a significant increase in spontaneous ex vivo fibrin clot degradation was noticed. According to Zuo et al. [23], the increased fibrinolytic activity in COVID-19 patients is influenced by various factors. SARS-Cov-2 virus exploits host-derived extracellular proteases, including plasmin, to efficiently infect cells. Plasmin enhances the virulence and infectivity of SARS-CoV-2 by cleaving its spike proteins.

Another mechanism that may explain the pathological fibrinolysis during the initial wound healing could be related to local events due to the COVID-19 infection. The local endothelial cells in the healing wound and surrounding gingival tissues could have been over-activated by the viral load of SARS-CoV-2. This over-activation of endothelial cells by SARS-CoV-2 can result in a high production of tPA, as has been described before [36]. Thus, we could speculate that the local endothelial cells in the damaged blood vessels of the wound while already in an activated state due to the periodontal surgery itself and the initial healing process produced increased levels of tPA. Therefore, the overproduction of tPA would further catalyze the conversion of plasminogen into plasmin, resulting in the premature degradation of the blood clot.

Our patient could also have had a thrombocytopenia at the time of the active COVID-19 infection simultaneously when we performed the third periodontal surgical procedure. Recently, a case series and literature review published in *British Journal of Haematology* [37] presented thrombocytopenia as an initial manifestation of COVID-19. In this case series, the second of the three patients described was a 49-year-old female with no known prior comorbidities who presented to her general practitioner as having had generalized bruises and gum bleeding for the previous three days. Her systemic examination was unremarkable, but laboratory results were suggestive of severe thrombocytopenia. The patient was also swabbed for COVID-19 and the result was positive for SARS-CoV-2 RNA detected by RT-PCR. In general, hematological manifestations in symptomatic COVID-19 patients include mild thrombocytopenia [38] with platelet counts ranging between 100 and 150 × 10^9^/L. Nevertheless, we do not consider thrombocytopenia in our case the underlying mechanism for the post-operative bleeding one day after the surgical procedure, since the initial blot-clot formation was normal and the post-operative bleeding occurred one day after the procedure. Moreover, the patient reported a lack of bruises in the period of interest.

The possibility of increased levels of tPA being at the base of the current post-operative bleeding event indicates that further study of tPA as a biomarker is needed in order to understand its exact role in the complex mechanism of the fibrinolytic homeostasis of this subset of COVID-19 patients. In addition, the increased fibrin degradation product D-dimer can be a common feature due to plasmin-associated hyperactive fibrinolysis in COVID-19 patients [39]. Compared to the group of patients in the study by Zuo et al. [23], the patient in the current case report exhibited a short period of an acute disease phase of COVID-19 without the need for hospitalization. Therefore, we have no data on any of the aforementioned blood plasma markers. We consider this is a limitation that leads only to the speculation of the cause of the observed post-operative bleeding.

Two months after the post-operative bleeding event, the patient underwent the fourth periodontal surgery. Here again the periodontal tissues of the patient exhibited excellent healing responses. The good healing potential of the patient was observed already at six months after the initial treatment, where several angular defects showed spontaneous bone fill, a phenomenon that is not noticed in every patient with intrabony defects. In addition, the substantial bone fill of the angular defects treated with amelogenin (enamel matrix derivative) during the surgeries attested to the good healing capacity of the patient. Therefore, the excellent healing of the patient in the initial phase of our treatment and the long-term results after the surgeries testify to the lack of co-morbidities, except for the acute COVID-19 phase.

## 4. Conclusions

After considering the normal response to the initial periodontal treatment and the well-documented good healing pattern of the periodontal tissues postoperatively for this patient with a free medical anamnesis, we conclude that the abnormal postoperative bleeding tendency was associated with an active phase of COVID-19. Clinicians should be aware that during the first postoperative days after a periodontal or oral surgery, patients with a postoperative bleeding event could have an underlying COVID-19 infection. The exact mechanism to explain the fibrinolysis is still to be determined.

Therefore, our concluding recommendations to clinicians and dental surgeons are:

(I) To meticulously interview the patient before the start of a surgical procedure regarding possible COVID-19-related complaints.
(II) In the event where a patient presents with general malaise shortly after the operation, she/he should be encouraged to have a PCR COVID-19 test, and if positive should be informed of possible intraoral bleeding complications and have a blood test performed as soon as possible regarding the coagulation state (biomarkers such as number of thrombocytes, tPA, uPA, PAI-1, and D-dimer).
(III) In the event of abnormal blood clot, the patient should be seen urgently in the clinic and extra suturing of the tissues should be decided on, as well as the application of pressure at the wound and instructions to the patient to rinse with tranexamic acid two to four times a day for two days.
(IV) In the case of a patient with an active COVID-19 infection where an oral surgical procedure cannot wait, immediately before the surgical intervention a blood test should be performed to test the coagulation state of the patient (see recommendation II).

## Data Availability

Not applicable.
